# Interpretable multimodal PET/CT-EHR fusion via mixture-of-experts for prognostic stratification in mantle cell lymphoma: a multicenter study

**DOI:** 10.1186/s12916-026-04865-1

**Published:** 2026-04-16

**Authors:** Chong Jiang, Zitong Zhang, Zekun Jiang, Chongyang Ding, Yue Teng, Limin Gao, Ming Jiang, Linhao Qu, Rong Tian

**Affiliations:** 1 Department of Nuclear Medicine and Clinical Nuclear Medicine Research Lab, West China Hospital, Sichuan University, Chengdu, 610041 Sichuan China; 2 Department of Nuclear Medicine, West China Hospital, Sichuan University, Chengdu, Sichuan China; 3 West China Biomedical Big Data Center, West China Hospital, Sichuan University, Chengdu, China; 4 Department of Nuclear Medicine, the First Affiliated Hospital of Nanjing Medical University, Jiangsu Province Hospital, Nanjing, China; 5 Department of Nuclear Medicine, Nanjing Drum Tower Hospital, the Affiliated Hospital of Nanjing University Medical School, Nanjing, China; 6 Department of Pathology, West China Hospital, Sichuan University, Chengdu, China; 7 Department of Oncology, West China Hospital of Sichuan University, Chengdu, China; 8 Shanghai Medical College, Fudan University, Shanghai, China

**Keywords:** Mantle cell lymphoma, PET/CT, Radiomics, Deep learning, Multimodal fusion, Prognostic stratification

## Abstract

**Background:**

Mantle cell lymphoma (MCL) is a rare, biologically heterogeneous B-cell malignancy with highly variable outcomes. Existing prognostic tools are suboptimal. We developed an interpretable deep learning framework integrating baseline [^18^F]FDG PET/CT and electronic health record (EHR) data for individualized risk stratification.

**Methods:**

In this multicenter study, 187 treatment-naïve MCL patients were analyzed. A mixture-of-experts (MoE) fusion network integrated multimodal representations from PET/CT and EHR data. Expert modules comprising vision encoders, radiomics extractors, and a medical language model were integrated through an attention-based gating mechanism to construct multimodal radiomic signatures (R-signatures) predictive of progression-free survival (PFS) and overall survival (OS). R-signatures were validated and incorporated with clinical and metabolic factors into multiparametric models. Deep learning model interpretability was evaluated using attention visualization, expert-level contributions and pathologic correlation.

**Results:**

R-signatures robustly discriminated relapse (AUC = 0.893 training, 0.755 validation) and death (AUC = 0.804 and 0.844), and independently predicted adverse outcomes (PFS: HR = 27.70, *P* < 0.001; OS: HR = 6.86, *P* = 0.001). Multiparametric models integrating R-signatures with total lesion glycolysis, β2-microglobulin, WBC, and Ki-67 outperformed conventional indices (C-indices: PFS 0.892 training, 0.781 validation; OS 0.877 training, 0.862 validation). Time-dependent ROC analyses consistently showed AUCs approaching or exceeding 0.800. Calibration and decision curve analyses confirmed excellent agreement and superior clinical net benefit. Attention maps localized high-weighted regions to hypermetabolic tumor areas, with higher R-signature values in blastoid and pleomorphic variants versus classical histology (*P* = 0.028 and *P* = 0.010).

**Conclusions:**

This interpretable PET/CT-EHR fusion framework substantially improves prognostic precision in MCL, providing a noninvasive, clinically translatable tool for risk-adapted management.

**Supplementary Information:**

The online version contains supplementary material available at 10.1186/s12916-026-04865-1.

## Background

Mantle cell lymphoma (MCL) is a rare subtype of B-cell non-Hodgkin lymphoma, characterized by the clonal proliferation of mature, antigen-experienced B lymphocytes, and accounts for approximately 5–7% of all non-Hodgkin lymphoma cases [1]. MCL is characterized by a heterogeneous clinical course, with patients exhibiting aggressive disease facing a markedly poor prognosis and a 5-year overall survival (OS) rate of only about 65% [2, 3]. Consequently, treatment strategies are increasingly personalized, combining intensive chemoimmunotherapy, autologous stem cell transplantation, and novel targeted agents such as BTK inhibitors to improve outcomes [4]. Effective risk stratification is therefore essential to guide these individualized therapies and enhance clinical prognosis.

The Mantle Cell Lymphoma International Prognostic Index (MIPI), which incorporates age, performance status, lactate dehydrogenase levels, and leukocyte count, is commonly used for risk stratification [5]. The Ki-67 proliferation index is combined with MIPI to form the biological MIPI (MIPI-c), which further improves prognostic precision [6]. However, predictive accuracy remains limited, particularly in identifying high-risk patients with poor prognosis, and challenges persist in accurately forecasting outcomes.

Positron emission tomography/computed tomography (PET/CT) using 18F-fluorodeoxyglucose (FDG) is routinely used for staging and response assessment in MCL [7]. Metabolic tumor burden indicators, such as total metabolic tumor volume (TMTV) and total lesion glycolysis (TLG), derived from baseline PET/CT scans, have shown significant prognostic value and serve as independent predictors of survival outcomes in mantle cell lymphoma [8, 9]. Nevertheless, PET-derived volumetric metrics may not fully capture the intratumoral heterogeneity underlying treatment resistance and relapse. Moreover, they insufficiently leverage the structural information available from CT, limiting a comprehensive evaluation of tumor biology.

Deep learning has substantially advanced oncologic prognostic modeling by integrating multimodal medical data like PET/CT imaging and electronic health record (EHR) data [10, 11]. However, several challenges continue to hinder the clinical translation of multimodal risk stratification. First, most existing approaches still analyze PET/CT and EHR data independently or integrate them using simplistic fusion strategies (e.g., late fusion or direct feature concatenation) [12–17]. Such methods often dilute modality-specific information and overlook critical cross-modal associations, particularly the links between three-dimensional metabolic–anatomic patterns on PET/CT and the semantic richness of clinical narratives. In addition, PET/CT and EHR data are inherently heterogeneous [10, 11]. PET/CT captures spatially resolved 3D representations of tumor metabolism and anatomy, whereas EHRs contain longitudinal, domain-specific textual information. Developing end-to-end frameworks capable of fully leveraging both modalities remains technically demanding, frequently resulting in inadequate feature extraction, unstable training, and suboptimal predictive performance. Finally, model interpretability remains limited [11, 18, 19]. Enhancing clinical explainability and aligning model predictions with other critical clinical information, such as pathological or molecular data, remain a major unmet need. These limitations underscore the urgent requirement for a unified multimodal framework capable of preserving modality-specific representations, effectively integrating heterogeneous data, and generating transparent, clinically interpretable explanations to advance precision oncology and personalized prognosis.

In this study, we propose a multimodal, multi-expert framework that integrates baseline PET/CT imaging and EHR data as equally weighted inputs to achieve systematic fusion for risk stratification in MCL. Moreover, the framework offers clear interpretability: PET/CT attention maps visualize risk-contributing regions; the learned mixture weights quantify the relative contribution of each expert modality; and pathology-consistent explanations are provided where applicable.

## Methods

### Patient data

**Fig. 1:**
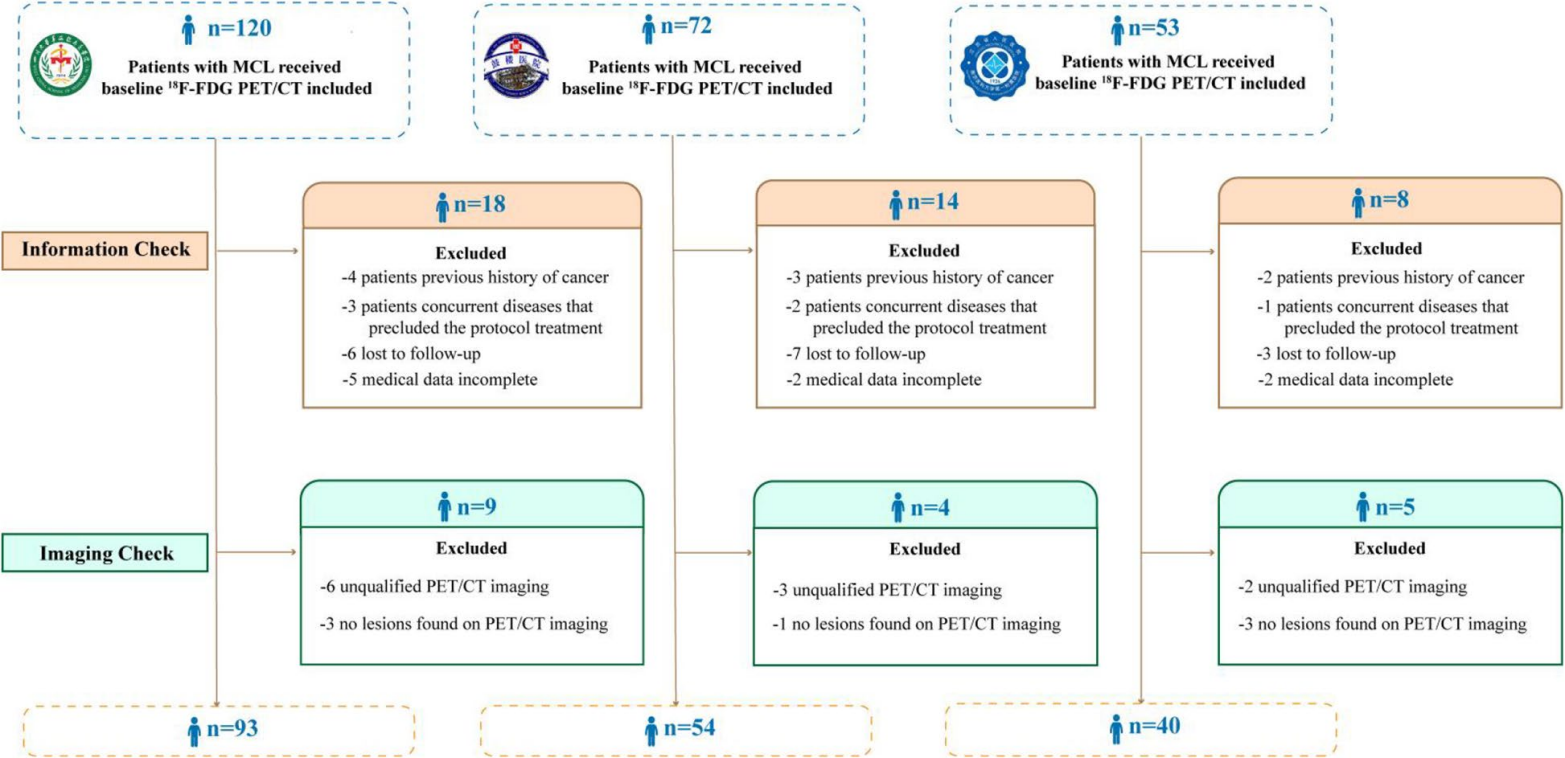
Flow chart of participant selection

In this multicenter study, patients from three independent medical centers were included. The patient population included 93 patients from West China Hospital, Sichuan University, 40 patients from Jiangsu Province Hospital, the First Affiliated Hospital of Nanjing Medical University, and 54 patients from Nanjing Drum Tower Hospital, the Affiliated Hospital of Nanjing University Medical School. Inclusion criteria required (1) a confirmed MCL diagnosis via histopathological examination; (2) completion of baseline [^18^F]FDG PET/CT scans; (3) no history of other tumors; and (4) availability of comprehensive medical records and follow-up information. Exclusion criteria encompassed (1) prior history of other tumors; (2) previous chemotherapy, radiation, or surgical treatment; (3) incomplete medical records; and (4) loss to follow-up. The detailed inclusion and exclusion criteria are provided in Fig. 1. This study was approved by the institutional review boards of each hospital involved (approval in 2024, No. 1384), which waived the need for written informed consent. Data sharing and protection policies were strictly adhered to, including anonymization of participant information to ensure privacy and confidentiality. The clinical information and follow-up data were obtained from electronic medical records and telephone interviews. After pooling data from the three centers, the entire cohort was randomly partitioned in an 8:2 ratio into a training cohort and a held-out validation cohort. The training cohort was used for model development and hyperparameter optimization, whereas the validation cohort was reserved exclusively for performance evaluation.

### PET/CT scanning protocol

All patients fasted for at least 6 h before scans, resulting in blood glucose levels under 8.7 mmol/L. Then, 185–370 MBq of [^18^F] FDG (5.18 MBq/kg) was administered intravenously. The PET/CT scans (from the base of the skull to the upper thigh) were performed 60 min after the radiopharmaceutical injection. Emission data were acquired for 2 min in each bed position. All patients underwent PET/CT scans with one of the following systems: Gemini GXL(reconstruction method: OSEM, PET slice thickness: 4 mm and PET pixel spacing: 4 mm), UM780PET/CT(reconstruction method: OSEM, PET slice thickness: 2.76 mm and PET pixel spacing: 2.9 mm), GE Discovery PET/CT clarity 710 (reconstruction method: VUE point FX, PET slice thickness: 3.75 mm and PET pixel spacing: 5.47 mm), and Biograph 16 PET/CT (reconstruction method: OSEM, PET slice thickness: 5 mm and PET pixel spacing: 4 mm). CT acquisition data were used for attenuation correction.

### Framework overview

**Fig. 2:**
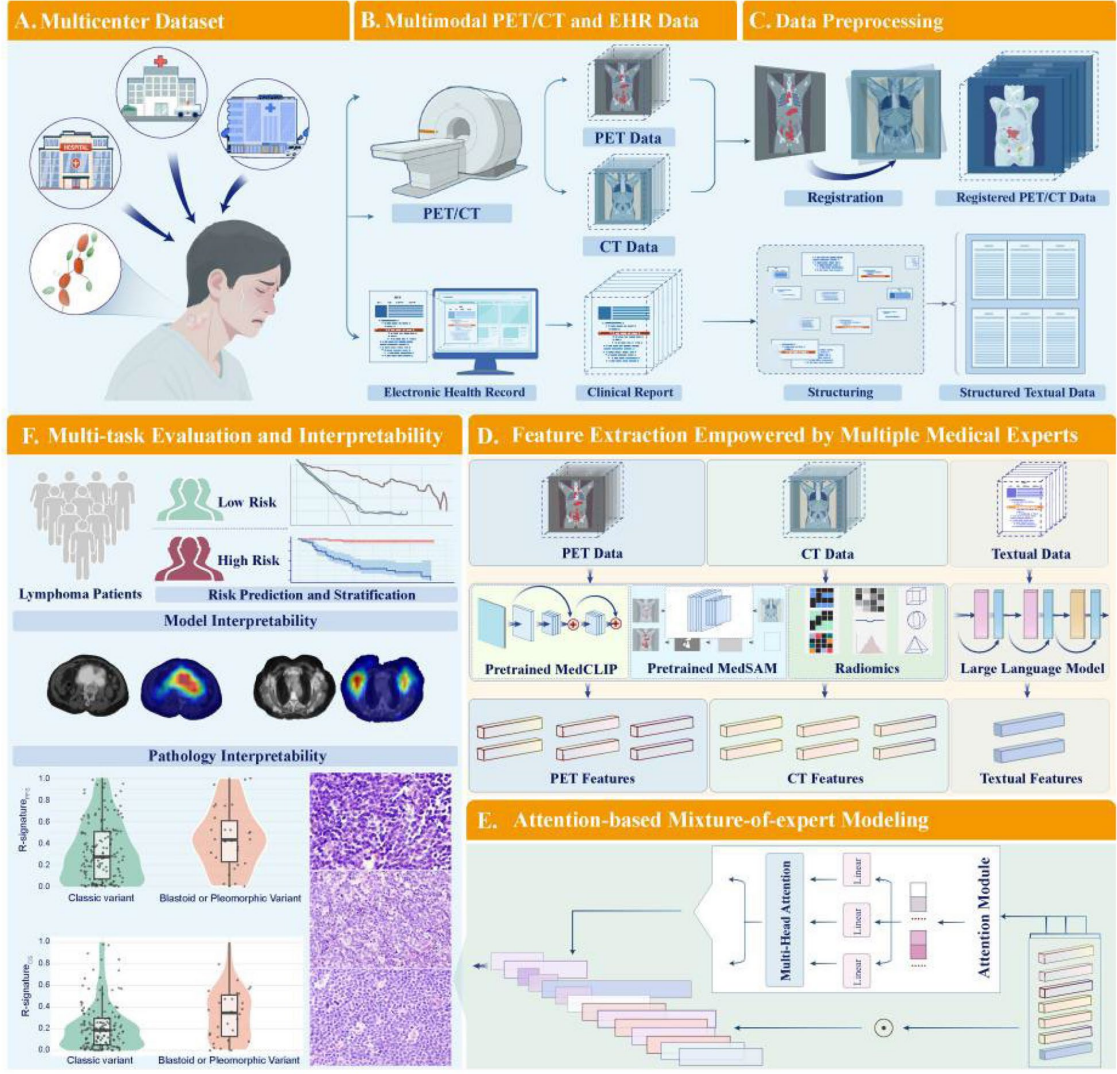
The workflow of this study

Figure 2 outlines the full workflow. We built a multimodal cohort in which each patient has paired PET/CT imaging and associated electronic health records (EHR; clinical notes and reports). The pipeline has four stages: (i) multimodal data acquisition, (ii) data preprocessing, (iii) expert-empowered feature extraction, and (iv) attention-based mixture-of-experts (MoE) modeling followed by a survival prediction layer (Additional file 1: Detailed Method).

### Multimodal alignment and structuring

#### Imaging

We developed an automatic PET-CT registration procedure that produces spatially aligned volumes for every study. PET and CT were resampled to a common voxel grid and clipped to modality-appropriate intensity ranges. PET uptake values were converted to standardized uptake values (SUV) and normalized within a robust body mask. To mitigate potential variability introduced by different PET/CT scanners and reconstruction algorithms across centers, standardized preprocessing procedures were applied prior to model development, including voxel resampling and modality-specific intensity normalization. These harmonization steps help reduce scanner-related variability and improve the stability and robustness of downstream feature representations. A rigid-affine registration aligned PET to CT by maximizing normalized mutual information while constraining extreme deformations. The result is a pair of registered PET and CT volumes per patient, which are also decomposed into ordered axial slices for downstream processing (Additional file 1: Detailed Method).

#### HER

Clinical reports were de-identified and segmented into sentences using a rule-augmented tokenizer with section detection (e.g., “Impression,” “Findings”). We applied negation and temporality tagging to each sentence and removed boilerplate text. All intermediate imaging and text outputs were reviewed by board-certified physicians. The final preprocessed bundle for each patient consists of a registered PET volume, a registered CT volume, and a structured, time-stamped set of clinical sentences (Additional file 1: Detailed Method).

### Data preprocessing

Two nuclear medicine physicians (each with > 5 years of experience) delineated tumor regions on PET images using LIFEx (version 7.3.0; https://www.lifexsoft.org/) [20]. Semi-automatic contours were generated with the 41% SUVmax threshold method on [^18^F]FDG PET images [21]. A senior nuclear medicine expert (> 10 years of experience) reviewed and confirmed all volumes of interest (VOIs). These annotations served as spatial references for subsequent feature extraction in model development.

### Expert-empowered feature extraction

We combined complementary “experts” across vision and language to extract feature groups from each modality. Each expert’s native outputs were projected to a shared embedding dimension using a single-layer multilayer perceptron (MLP), enabling unified downstream modeling.

*Discriminative vision expert (MedCLIP) for PET/CT* [22]: For every PET and CT slice, MedCLIP produces a semantic embedding that captures high-level visual context (e.g., tumor versus background patterns).

*Segmentation-centric vision expert (MedSAM) for PET/CT* [23]*.* For the same slices, MedSAM yields morphology-oriented embeddings focused on lesion shape, size, boundaries, and local spatial context.

*Classical radiomics for PET/CT* [24]: Standardized radiomic features were computed per slice and then projected to the shared embedding space.

*Language expert (Med-BERT) for clinical text*: Each clinical sentence was embedded with a domain-adapted medical language model and projected to the shared space.

Embeddings were organized into expert-specific groups that preserve inductive biases (e.g., one group for MedCLIP-CT, one for MedCLIP-PET, etc., and one for Med-BERT text). This grouping enables targeted attention within and across modalities. Specifically, after PET/CT registration, each aligned PET slice and CT slice was processed independently by MedCLIP and MedSAM, generating four deep feature groups: MedCLIP-PET, MedCLIP-CT, MedSAM-PET, and MedSAM-CT. Handcrafted radiomic features were extracted separately from the PET VOI and the corresponding CT VOI, yielding Radiomics-PET and Radiomics-CT. Clinical text was encoded by Med-BERT to form a seventh expert group. All seven groups were projected to a common embedding dimension before fusion (Additional file 1: Detailed Method).

### Attention-based mixture-of-experts (MoE) fusion

Our fusion network proceeds in two symmetric stages.

#### Intra-group aggregation

Within each expert group, a lightweight Transformer encoder applies multi-head self-attention to capture relationships among the group’s tokens (e.g., slices or sentences). The encoder output is then summarized into a single vector per group using learned attention pooling with a global query. This step yields one compact representation for each expert group (e.g., MedCLIP-CT, MedCLIP-PET, MedSAM-CT, MedSAM-PET, Radiomics-CT, Radiomics-PET, and Med-BERT-Text) (Additional file 1: Detailed Method).

Specifically, for expert group $g$ with token sequence ${X}^{\left(g\right)}=\{{\mathbf{x}}_{i}^{\left(g\right)}{\}}_{i=1}^{{n}_{g}}$, we compute contextualized features ${H}^{\left(g\right)}={E}_{\mathrm{intra}}^{\left(g\right)}\left({X}^{\left(g\right)}\right)=\{{\mathbf{h}}_{i}^{\left(g\right)}{\}}_{i=1}^{{n}_{g}}$. A learned query ${\mathbf{q}}^{\left(g\right)}$ generates pooling weights:

$$\alpha_i^{\left(g\right)}=\frac{\exp\left(\left(\mathbf q^{\left(g\right)}\right)^{\mathrm T}\mathbf h_i^{\left(g\right)}\right)}{\sum_{j=1}^{n_g}\exp\left(\left(\mathbf q^{\left(\mathrm g\right)}\right)^{\mathrm T}\mathbf h_j^{\left(g\right)}\right)}$$

and the group representation is:

$${\mathbf{z}}^{\left(g\right)}=\sum_{i=1}^{{n}_{g}}{\alpha }_{i}^{\left(g\right)}\hspace{0.17em}{\mathbf{h}}_{i}^{\left(g\right)}$$

#### Inter-group mixture with gating

All group vectors are passed through a cross-group Transformer to allow interactions between experts. A gating network then produces non-negative mixture weights over groups. The final decision embedding is a weighted combination of the refined group vectors, where the weights adaptively emphasize the experts most informative for the current patient.

Specifically, let $Z=\left[{\mathbf{z}}^{\left(1\right)},\dots ,{\mathbf{z}}^{\left(G\right)}\right]$ and $\widetilde{Z}={E}_{\mathrm{inter}}\left(Z\right)=\{{\widetilde{\mathbf{z}}}^{\left(g\right)}{\}}_{g=1}^{G}$. The gating network outputs scores $\mathbf{s}=\mathrm{Gate}\left(\widetilde{Z}\right)\in {\mathbb{R}}^{G}$, which are normalized as:

$${\pi }_{g}=\frac{\mathrm{exp}\left({s}_{g}\right)}{\sum_{k=1}^{G}\mathrm{exp}\left({s}_{k}\right)}, {\pi }_{g}\ge 0, \sum_{g=1}^{G}{\pi }_{g}=1$$

The final patient-level embedding is:

$$\mathbf{u}=\sum_{g=1}^{G}{\pi }_{g}\hspace{0.17em}{\widetilde{\mathbf{z}}}^{\left(g\right)}$$

### Survival prediction layer and task-specific training

We trained two independent survival models, one for overall survival (OS) and one for progression-free survival (PFS). Each task uses its own copy of the fusion module and a linear risk head with the same architecture but separate parameters. The task-specific fusion module produces a patient embedding that is mapped to a scalar risk score.

Each task is optimized only on its corresponding survival data (event times and censoring indicators) using the negative Cox partial log-likelihood. At inference, the OS model outputs the OS risk score, and the PFS model outputs the PFS risk score. Evaluation is performed separately for each endpoint (Additional file 1: Detailed Method).

### Assessment and validation of the multiparametric models

Multivariate analysis was used to identify significant clinical risk factors and PET metric, which were then employed with R-signatures to develop multiparametric models. The calibration curves were plotted for the models, and their discrimination capability was measured by computing Harrell’s C-index. Additionally, time-dependent ROC curve and decision curve analysis (DCA) were performed to evaluate the clinical net benefit of the models. Finally, the model’s performance was evaluated in validation cohorts.

### Interpretability analyses

To elucidate how the model arrives at patient-level risk estimates, we combine attention-based, expert-level, and clinicopathologic interpretability within a single framework. First, we propagate attention through the intra-group Transformers (attention rollout) to obtain slice-wise importance distributions for both PET and CT; these distributions are upsampled and overlaid on the registered volumes to yield anatomically faithful heatmaps that highlight regions exerting the greatest influence on the prediction. Second, the inter-group gating network furnishes calibrated mixture weights that quantify the contribution of each expert group (e.g., PET-CLIP semantics, CT-radiomics, MedSAM-derived morphology, or sentence-level EHR embeddings) to a given decision, thereby revealing modality-specific and expert-specific drivers on a case-by-case basis. Finally, we associate the resulting risk markers with specific histopathologic lymphoma subtypes, namely aggressive types (such as pleomorphic and blastoid) and classical types, by comparing model-defined risk strata across variants and covariates and by visualizing distributional shifts.

### Statistical analysis

The statistical analyses were performed using SPSS 22.0 (IBM Corp., Armonk, NY, USA) and R statistical software (version 4.0.2). Statistical significance was set at a P-value less than 0.05. Progression-free survival (PFS) and overall survival (OS) were employed as the endpoints to assess the prognosis of patients with MCL. Differences in clinical data between the training and testing cohorts were appraised using independent-sample *t*-tests, *χ*^2^ tests, or Mann–Whitney *U* tests. Standardized mean differences (SMDs) were calculated, with an SMD < 0.1 considered to indicate a well-balanced distribution between groups. The optimal threshold values for the SUVmax, TMTV, TLG, and R-signatures were determined using the receiver operating characteristic (ROC) curve in the training cohort. Cox regression analysis was used to explore the prognostic value of potential independent factors and construct models. Survival was evaluated by the Kaplan–Meier method and compared by the log-rank test.

## Results

### Patient characteristics

The characteristics of patients in the training cohort and the validation cohort are summarized in Table 1. No statistically significant differences were observed in any clinical variables between the two cohorts (*P* > 0.05; Table 1). Median follow-up periods for the training cohort and the validation cohort were 33.0 and 27.5 months, respectively. In the training cohort, disease relapse occurred in 39 participants, and 20 participants died. In the validation cohort, 21 participants experienced disease relapse or progression, and 9 participants died.

**Table 1:** Demographics and characteristics of the study population

Characteristic	Total (*n* = 187)	Training cohort (*n* = 125)	Validation cohort (*n* = 62)	*P* value	SMD
Sex, male/female	141/46	96/29	45/17	0.652	0.097
Age, < 60/≥ 60	78/109	52/73	26/36	1.000	0.007
Ann Arbor stage (I–II/III–IV)	25/162	16/109	9/53	0.923	0.05
B symptoms, yes/no	64/123	46/79	18/44	0.373	0.166
Splenomegaly, yes/no	59/128	37/88	22/40	0.517	0.126
Bulky disease, yes/no	23/164	17/108	6/56	0.594	0.123
ECOG PS, ≥ 2/0–1	10/177	6/119	4/58	0.899	0.072
LDH, elevated/normal	45/142	31/94	14/48	0.879	0.052
β2-MG, elevated/normal	96/91	63/62	33/29	0.835	0.057
WBC, elevated/normal	36/151	23/102	13/49	0.824	0.065
Bone marrow involvement, yes/no	80/107	54/71	26/36	0.994	0.026
Ki-67, high/low	86/101	56/69	30/32	0.758	0.072
Treatmet, BTKi-based/R-CHOP like	62/125	42/83	20/42	0.985	0.029
MIPI, low-risk/intermediate-risk/high-risk	68/78/41	41/50/34	27/28/7	0.258	0.367
MIPI-c, low-risk/low-intermediate-risk/high-intermediate-risk/high-risk	36/71/63/17	21/48/41/15	15/23/22/2	0.173	0.255

Abbreviations: *LDH* Lactate dehydrogenase, *ECOG PS* Eastern Cooperative Oncology Group performance status, *β2-MG* β2-microglobulin, *WBC* White blood cell count, *SMD* Standardized mean difference

^*^P < 0.05

### Predictive performance of the R-signatures for PFS and OS

**Fig. 3:**
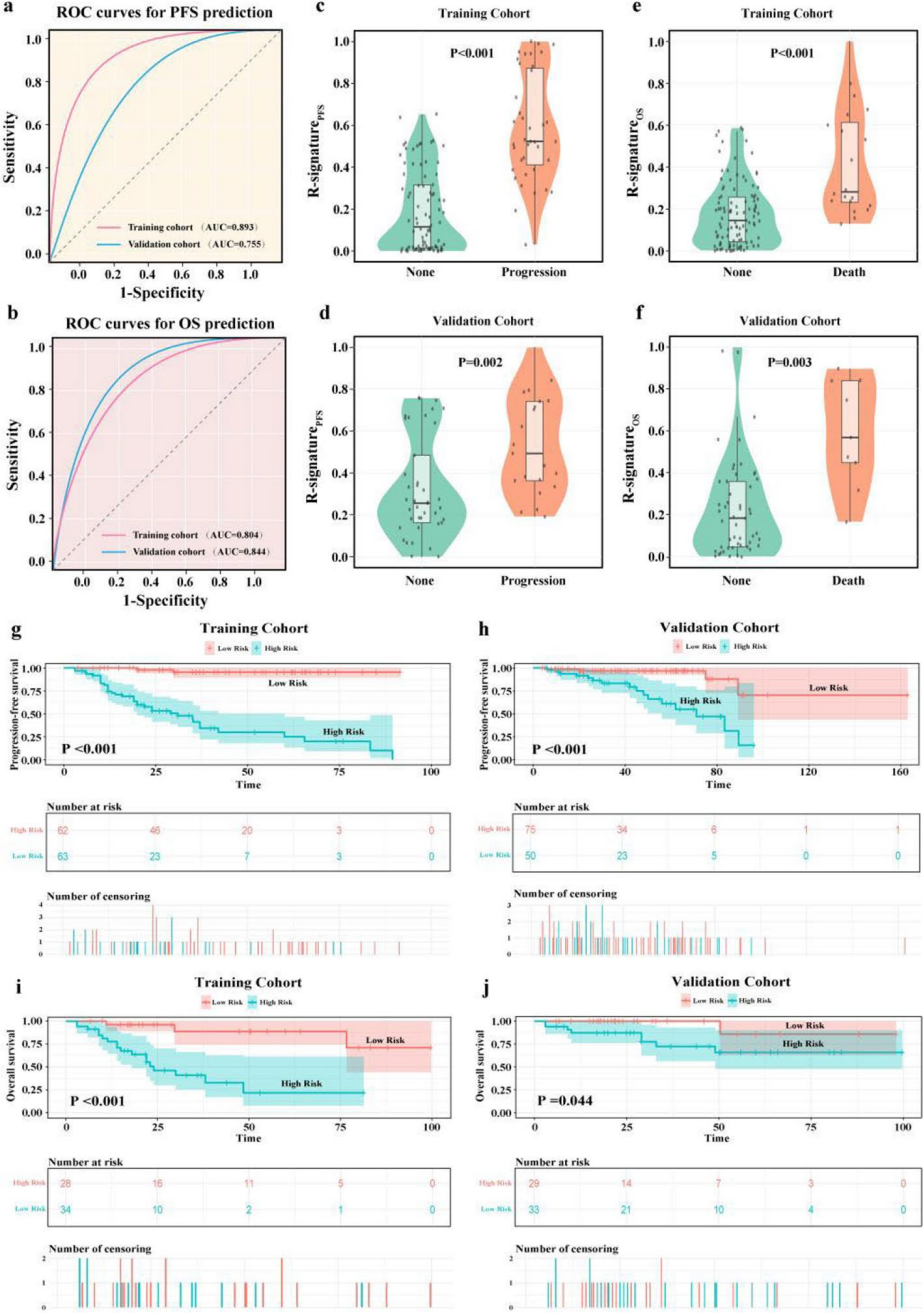
Predictive performance of R-signatures for PFS and OS. **a**–**b** ROC curves and violin plots for PFS and OS prediction in the training and validation cohorts. **c**–**f** Violin plots compare R-signature levels between patients with and without events (progression or death). **g**–**j** Kaplan–Meier survival curves for PFS and OS in the training and validation cohorts, stratified by high-risk and low-risk groups based on R-signatures

For both PFS and OS prediction (Fig. 3), the R-signature demonstrated strong discriminatory performance. The area under the ROC curve (AUC) was 0.893 and 0.804 in the training cohort, and 0.755 and 0.844 in the validation cohort for PFS and OS, respectively (Fig. 3). Violin plots showed that R-signature levels were significantly higher in patients who experienced progression or death compared with those without these events in both cohorts (*P* < 0.001 for PFS and OS in the training cohort; *P* = 0.002 for PFS and *P* = 0.003 for OS in the validation cohort) (Fig. 3). Kaplan–Meier survival analysis further confirmed the prognostic value of the R-signature. High-risk patients stratified by R-signature had significantly shorter PFS and OS than low-risk patients in the training cohort (*P* < 0.001 for both) and in the validation cohort (*P* < 0.001 for PFS and *P* = 0.044 for OS) (Fig. 3). The optimal cut-off values for traditional PET parameters were 27.1 for SUVmax, 133.1 cm^3^ for TMTV, and 788.6 for TLG.

### Results of univariate and multivariate analyses

**Fig. 4:**
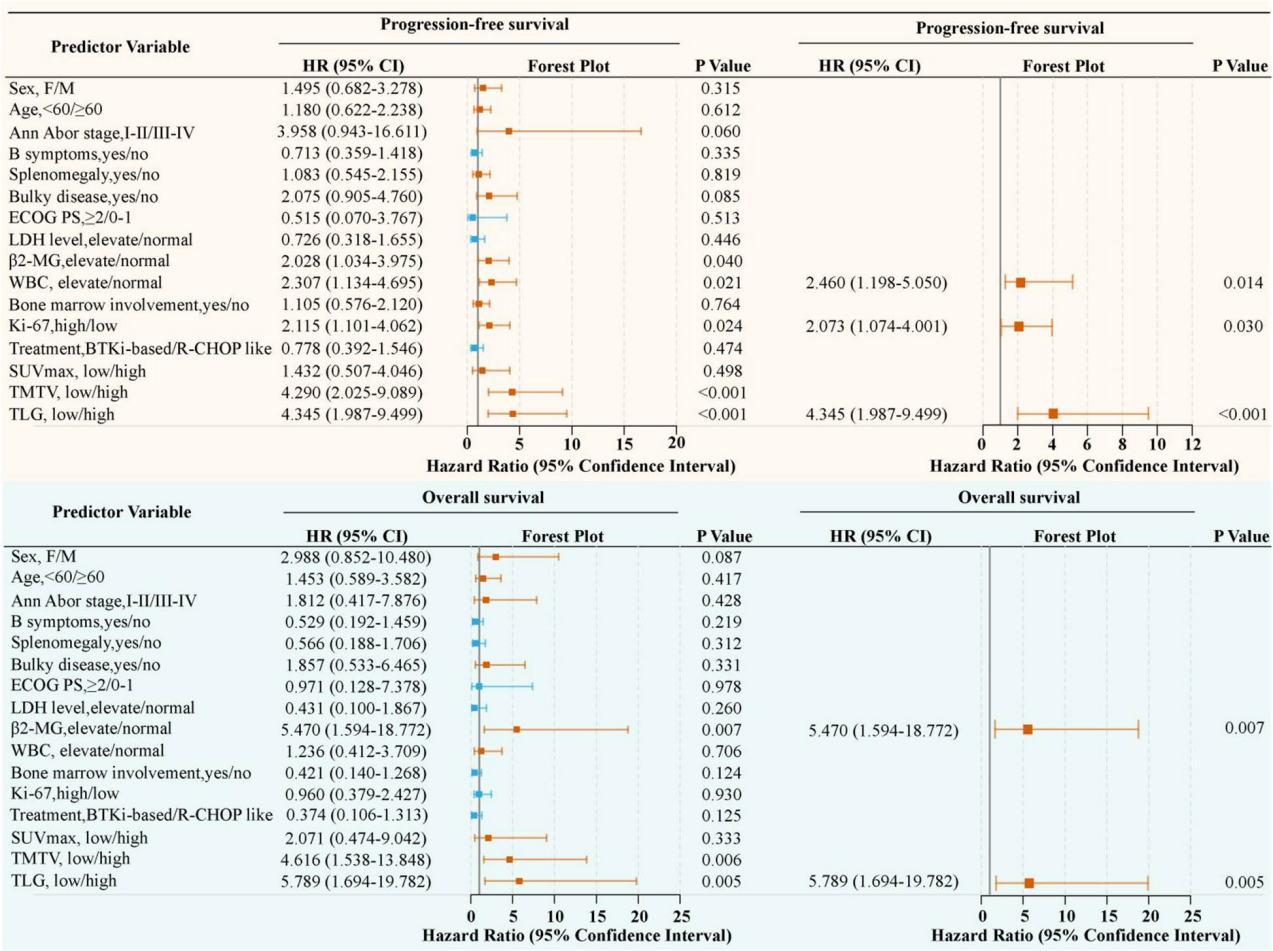
Forest plot showing underlying predictors of PFS and OS based on univariate and multivariate analyses results

Univariate and multivariate analyses revealed that among clinical variables, elevated β2-MG was an independent predictor of OS (HR = 5.470, *P* = 0.007). Moreover, increased WBC (HR = 2.460, *P* = 0.014) and higher Ki-67 expression (HR = 2.073, *P* = 0.030) were independently associated with poorer PFS. Among PET-derived parameters, TLG emerged as a robust independent prognostic factor for both PFS (HR = 4.345, *P* < 0.001) and OS (HR = 5.789, *P* = 0.005). A detailed summary of these results is presented in Fig. 4 and Additional file 2: Table S1. In addition, the R-signatures demonstrated strong and independent prognostic significance for both endpoints—PFS (HR = 27.702, *P* < 0.001) and OS (HR = 6.862, *P* = 0.001) (Additional file 2: Table S1).

### Assessment and validation of the multiparametric models

**Fig. 5:**
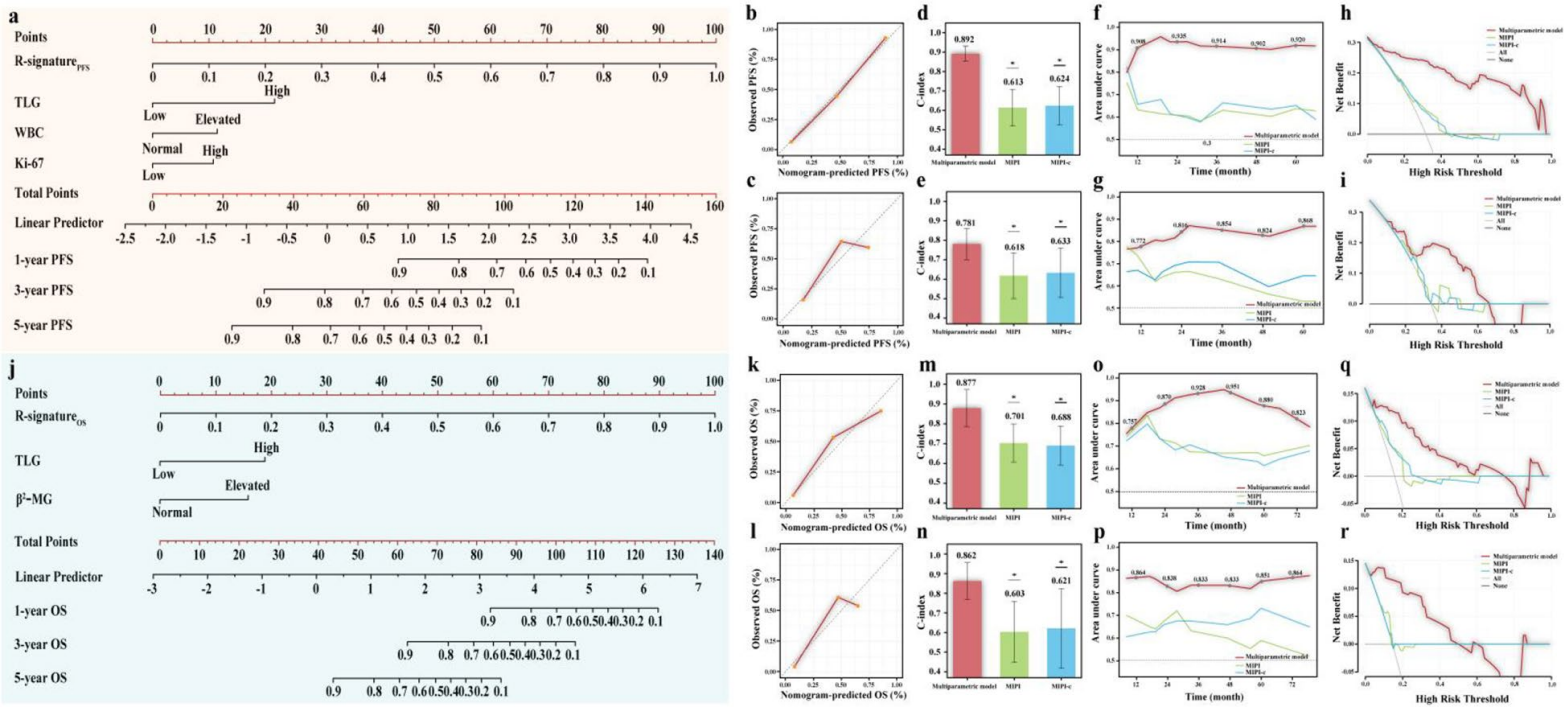
Performance of the multiparametric models for PFS and OS. (a) The multiparametric nomogram for predicting PFS. (b-c) Calibration curves of the model for predicting PFS in the training and validation cohorts. **d**–**e** Bar plots comparing the C-index between the multiparametric PFS model and existing prognostic systems in the training and validation cohorts. **f**–**g** The time-dependent area under the ROC curve of the models for predicting PFS in the training and validation cohorts. **h**–**i** Decision curve analysis of the models for predicting PFS in the training and validation cohorts. **j** The multiparametric nomogram for predicting OS. **k**–**l** Calibration curves of the model for predicting OS in the training and validation cohorts. **m**–**n** Bar plots comparing the C-index between the multiparametric OS model and existing prognostic systems in the training and validation cohorts. **o**–**p** The time-dependent area under the ROC curve of the models for predicting OS in the training and validation cohorts. **q**–**r** Decision curve analysis of the models for predicting OS in the training and validation cohorts

Multiparametric prognostic models were developed by integrating R-signatures, metabolic parameters (TLG), and clinical variables (WBC and Ki-67 for PFS; β2-microglobulin for OS) (Fig. 5). Calibration curves demonstrated good agreement between predicted and observed outcomes across both the training and validation cohorts. The models showed strong discriminative performance, with C-indices of 0.892 (95% CI: 0.853–0.931) for PFS and 0.877 (95% CI: 0.783–0.971) for OS in the training cohort, and 0.781 (95% CI: 0.701–0.861) for PFS and 0.862 (95% CI: 0.768–0.956) for OS in the validation cohort—consistently outperforming conventional models such as MIPI and MIPI-c (Fig. 5 and Additional file 2: Table S2).

Time-dependent ROC analysis further confirmed the superior prognostic accuracy of the multiparametric models. In the training cohort, the AUCs for PFS at 1, 3, and 5 years were 0.908, 0.914, and 0.920, respectively, and for OS were 0.870, 0.951, and 0.823. In the validation cohort, the corresponding AUCs were 0.772, 0.854, and 0.868 for PFS, and 0.864, 0.833, and 0.864 for OS (Fig. 5).

Additionally, DCA demonstrated greater net clinical benefit of the multiparametric models across a wide range of risk thresholds for both endpoints, further supporting their utility over MIPI and MIPI-c (Fig. 5).

### Interpretability analysis

**Fig. 6:**
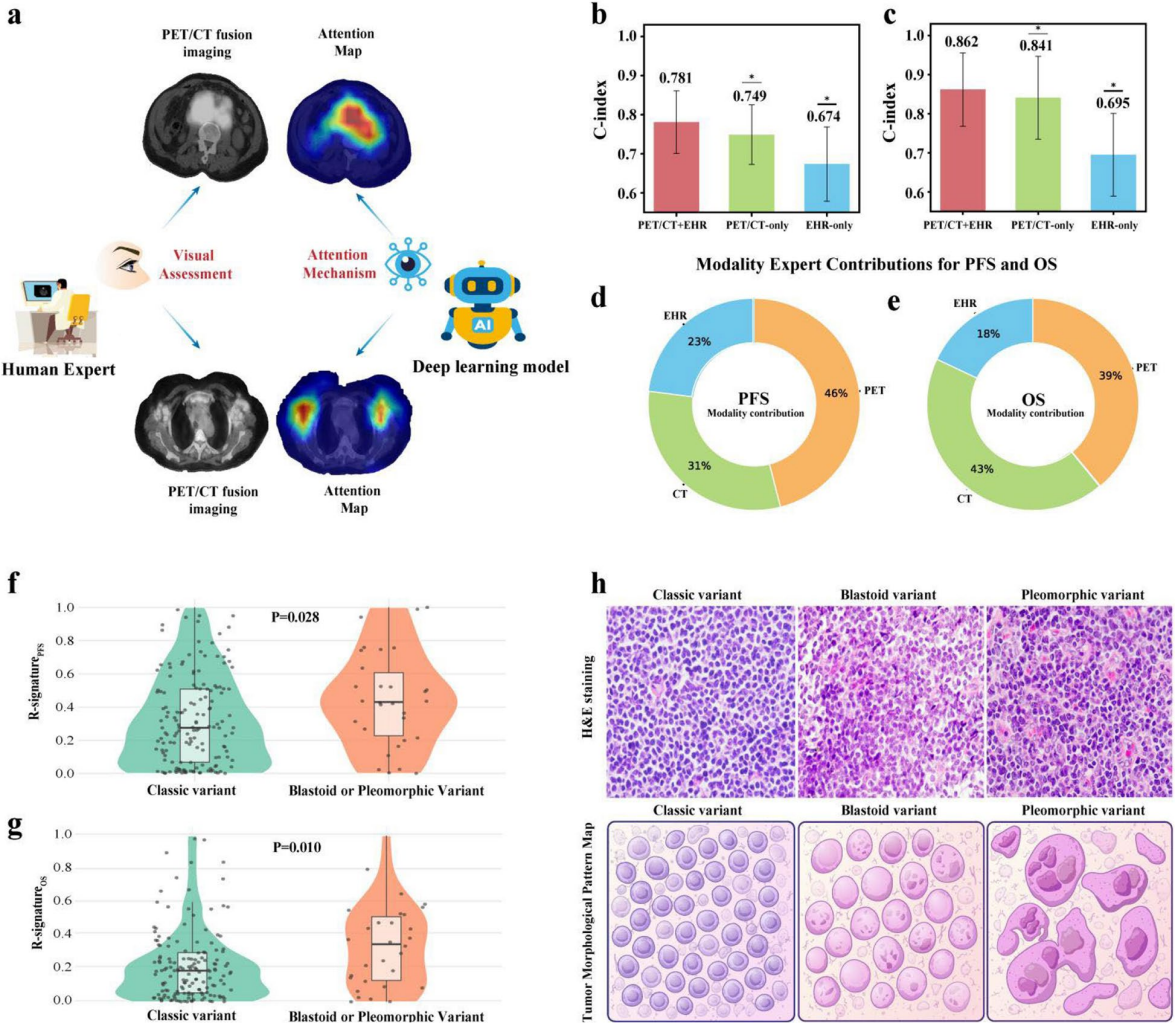
Interpretability of the deep learning model. **a** Representative PET/CT and corresponding attention heatmaps illustrate that the model primarily focuses on hypermetabolic tumor regions, with prominent attention concentrated in the abdominal and bilateral axillary lesions—patterns consistent with the areas typically evaluated by clinicians during visual assessment. **b**–**e** Quantitative analysis of modality-level contributions for PFS and OS, calculated by averaging inter-group gating weights across all patients. **d**–**c** Ablation study demonstrating the performance impact of each modality (PET/CT and EHR) on PFS and OS prediction, confirming the complementary value of multimodal fusion. **f**–**g** Violin plots show the distribution of R-signature values across histologic subtypes, revealing significantly higher scores in blastoid or pleomorphic variants compared with the classic variant (*P* = 0.028 and *P* = 0.010). **h** Representative hematoxylin–eosin (HE) staining images illustrating morphological differences: classic—small to medium lymphoid cells with irregular nuclei and nodular or mantle zone pattern; blastoid—intermediate, lymphoblast-like cells with dispersed chromatin and high mitotic activity; pleomorphic—large, irregular nuclei with prominent nucleoli, indicating more aggressive morphology

Interpretability analysis provided further insights into the multimodal model’s decision process and biological relevance (Fig. 6). The attention heatmaps demonstrated that the model consistently emphasized hypermetabolic tumor regions on PET/CT images, aligning with the areas typically assessed by clinicians. Quantitative analysis of the gating weights revealed that for OS, PET and CT contributed comparably, whereas for PFS, PET played a more dominant role. Incorporating EHR data yielded a modest but consistent improvement in discrimination, particularly for PFS, confirming the complementary value of multimodal fusion. Furthermore, the model-derived R-signature successfully captured histopathological heterogeneity. Patients with blastoid or pleomorphic variants exhibited significantly higher R-signature values than those with the classic variant (*P* = 0.028 and *P* = 0.010, respectively), suggesting that the model not only identifies metabolic and morphological features but also reflects underlying biological aggressiveness.

## Discussion

In this multicenter investigation, we developed and validated novel PET/CT-EHR-based multimodal signatures (R-signatures) capable of identifying patients at high risk of adverse prognosis. The R-signatures demonstrated strong predictive power for risk stratification, and when combined with metabolic and clinical variables, yielded highly robust multiparametric models for individualized prognostic assessment in MCL, providing a practical tool to support precision treatment decisions.

MCL is a biologically and clinically heterogeneous B-cell malignancy characterized by marked variability in therapeutic response and survival outcomes among patients [25]. Identifying individuals at high risk of progression or relapse who may benefit from treatment intensification remains a key unmet need [4]. Recent advances in artificial intelligence (AI) have highlighted its promise as a prognostic tool for optimizing treatment strategies, assessing remission, and predicting relapse across hematologic malignancies [26]. However, investigations focusing specifically on radiomics-derived imaging biomarkers from PET or PET/CT in MCL are still limited [27–29]. In a representative study involving 107 treatment-naïve MCL patients, [^18^F]FDG PET/CT-derived SUVmean and entropy were identified as significant predictors of 2-year progression-free survival (PFS), yielding an AUC of 0.82 when integrated with clinical and laboratory factors [27]. Beyond prognostic prediction, recent studies have demonstrated that [^18^F]FDG PET/CT-based radiomic features can also enhance disease characterization in MCL. For instance, texture features extracted from pelvic bone marrow improved the prediction of marrow involvement compared with conventional SUV metrics, achieving an AUC of up to 0.85 when combined with laboratory parameters [28]. Likewise, analysis of baseline PET/CT data from the prospective LyMa-101 trial revealed that radiomic features outperformed clinical and biological parameters in predicting minimal residual disease (MRD) status prior to autologous stem cell transplantation [29]. Consistent with these findings, our study demonstrated that radiomics-derived R-signatures from baseline PET/CT scans serve as robust and independent prognostic biomarkers for both PFS (HR = 27.702, *P* < 0.001) and OS (HR = 6.862, *P* < 0.001) in MCL. Collectively, our findings underscore the promise of AI-based PET/CT analysis as a powerful, noninvasive approach for identifying high-risk MCL patients who may benefit from therapeutic escalation or closer clinical surveillance, ultimately facilitating precision, risk-adapted management in this challenging malignancy.

Our study bridges critical gaps in multimodal prognostic modeling by coupling modality-specific expert networks with a rigorously designed fusion framework. Previous approaches have typically processed PET/CT and EHR data either in isolation or through naïve late fusion or feature concatenation, which can dilute modality-specific information and obscure important cross-modal relationships previously reported in the literature [12–17]. In contrast, our framework preserves the unique strengths of each modality by employing specialized experts that emulate clinical interpretive processes, with PET experts capturing metabolic heterogeneity, CT experts focusing on morphological characteristics, and a language expert encoding clinically meaningful textual information. These expert embeddings are integrated through a hierarchical, attention-guided mixture-of-experts mechanism: intra-group attention refines slice-level or sentence-level inputs into compact, noise-reduced embeddings, while a cross-group gating module dynamically assigns adaptive, patient-specific weights to each expert. By maintaining modality-specific representations and fusing them only when their latent features become comparable, this architecture effectively addresses the heterogeneity inherent in 3D imaging and clinical narratives. Consequently, it yields more stable model training and superior discriminative performance compared with late fusion or simple concatenation strategies. Further, we selected MedCLIP and MedSAM because they provide complementary inductive priors for PET/CT analysis: MedCLIP captures high-level semantic/contextual information from medical image-text pretraining, whereas MedSAM emphasizes lesion morphology and boundary-aware local structure. This pairing enables the MoE framework to combine semantic and morphology-sensitive cues while reducing overfitting risk in a modest-sized multicenter cohort.

Importantly, our multiparametric models outperformed MIPI and MIPI-c, which, despite their widespread adoption, do not fully capture the phenotypic diversity of MCL. By synergistically integrating functional imaging biomarkers, quantitative tumor burden, and clinical indicators of biological aggressiveness (β2-MG, WBC, and Ki-67), we constructed a framework that captures complementary dimensions of disease risk. Such a multidomain approach aligns with the evolving paradigm of precision oncology, in which molecular, imaging, and clinical data are jointly leveraged to guide treatment. Clinically, these models could refine therapeutic decision-making in MCL, enabling high-risk patients to be triaged toward intensified regimens or early integration of targeted therapies, while sparing low-risk patients from unnecessary treatment toxicity. Given that PET/CT is already embedded in the diagnostic workup of MCL, radiomics-based risk stratification could be seamlessly incorporated into current workflows without additional procedural burden. Intriguingly, our analysis did not identify the treatment regimen (BTKi-based vs. R-CHOP-like) as an independent prognostic factor for either PFS or OS (*P* > 0.05). This lack of statistical significance may be attributed to several factors. First, the median follow-up of 33 months in this cohort may be insufficient to fully capture the long-term survival advantage of targeted agents in MCL, where survival curves typically exhibit more pronounced divergence beyond 5 years. Second, selection bias remains a significant confounding factor in this retrospective study; clinicians may have preferentially assigned frailer patients to the BTKi-based regimen due to its more favorable toxicity profile, potentially masking the drug’s inherent therapeutic efficacy. Despite these limitations, the BTKi-based group demonstrated notable clinical trends toward improved outcomes in OS (HR = 0.374 for OS; mortality rate: 7.1% vs. 20.5%), suggesting that the therapeutic benefits of novel regimens may become statistically evident with extended longitudinal observation.

The framework improves interpretability at several levels. Spatial attention maps computed on co-registered PET/CT volumes produce anatomically coherent saliency patterns, highlighting disease-relevant regions rather than diffuse background signal. These maps, together with quantitative feature summaries, reveal subtype-specific imaging signatures in mantle cell lymphoma, supporting face validity for the model’s focus and offering hypotheses for biological heterogeneity. To further elucidate the pathological interpretability of the R-signature, we examined the quantitative distributions of model-defined risk across histopathologic subtypes of MCL. The quantitative R-signature values differed significantly between subtypes, with aggressive variants showing higher median scores than classical forms, consistent with their more adverse biological behavior. These findings indicate that the R-signature captures underlying histopathologic heterogeneity, providing a mechanistic link between noninvasive imaging features and the biological behavior of MCL, thereby enhancing both prognostic precision and model interpretability. We also quantified modality contributions by averaging the inter-group gating weights across patients for OS and PFS. PET and CT collectively accounted for most of the risk attribution, while EHR features contributed a smaller but non-negligible share. This weighting aligns with clinical expectations that structural and metabolic disease burden is central to risk assessment, with clinical context adding complementary nuance. Ablation analyses further support this interpretation. Models restricted to PET/CT clearly outperformed EHR-only models for both OS and PFS, indicating that imaging is the primary driver of discrimination. Yet incorporating EHR features consistently improved performance, with greater gains observed for PFS than for OS, consistent with the higher EHR weights learned for PFS. This pattern is biologically plausible: near-term progression is more sensitive to contemporaneous clinical factors (e.g., symptoms, treatment changes, and complications) that are captured in notes, whereas OS remains more strongly tied to the overall metabolic and structural disease burden seen on imaging. Together, these findings show that the model’s explanatory signals are coherent with clinical reasoning and that EHR data meaningfully complements imaging—particularly for predicting progression.

Despite these promising results, several limitations should be acknowledged. First, the overall sample size remains relatively modest, reflecting the rarity of MCL, and underscores the need for broader prospective validation in larger, multicenter, international cohorts. Second, radiomic features represent indirect surrogates of tumor biology; their prognostic value could be enhanced by integration with molecular biomarkers, particularly TP53 mutation status, a key determinant of aggressive disease and poor outcomes in MCL [25]. However, TP53 testing was not routinely available across all centers in this real-world cohort, and its inclusion would have introduced substantial missing data and potential selection bias. Notably, the model was intentionally designed as a noninvasive tool based on baseline PET/CT and routinely accessible clinical variables to ensure broad clinical applicability. Future prospective studies integrating genomic and imaging features are warranted to further improve biological interpretability and prognostic accuracy. Finally, variability in imaging acquisition and reconstruction parameters may affect feature reproducibility, highlighting the importance of standardized imaging protocols in future multi-institutional studies to ensure robust and generalizable results.

## Conclusions

In summary, we demonstrate that multimodal, interpretable risk signatures integrating PET/CT and EHR can enable risk-adapted management in MCL. This approach enhances prognostic stratification while maintaining interpretability, offering practical insights to inform precision oncology and treatment decisions.

Supplementary information.

## Supplementary Information

Additional file 1: Detailed Method. This file provides a comprehensive description of the methodological framework, including:framework overview;data preprocessing;feature extraction empowered by multiple medical experts;attention-based Mixture-of-Experts modeling; andsurvival prediction layer with task-specific optimization

Additional file 2: Tables S1-S2. Table S1—[Univariate analyses of factors predictive of progression-free survival and overall survival in the training cohort.]. Table S2—[The Harrell’s C-index results in the training and validation cohorts]

## Data Availability

The data that support the findings of this study are available from the cor responding author upon reasonable request. The underlying code for this study is available in https://github.com/Mr-Zeros/Multimodal-PET-CT-EHR-Fusion-for-Prognostic-Stratification-in-Mantle-Cell-Lymphoma/tree/main

## Abbreviations

MCL: Mantle cell lymphoma
EHR: Electronic health record
MoE: Mixture-of-experts
R-signature: Radiomic signature
MIPI: Mantle Cell Lymphoma International Prognostic Index
TMTV: Total metabolic tumor volume
TLG: Total lesion glycolysis
VOI: Volume of interest
DCA: Decision curve analysis
ROC: Receiver operating characteristic
PFS: Progression-free survival
OS: Overall survival
