# Supplementary material for: Interpretable multimodal PET/CT-EHR fusion via mixture-of-experts for prognostic stratification in mantle cell lymphoma: a multicenter study
Source: BMC Med. 2026 Apr 16;24:330. doi: 10.1186/s12916-026-04865-1 (PMC13202896; doi:10.1186/s12916-026-04865-1)
Supplement: Supplementary file 1 — Additional file 1: Detailed Method. This file provides a comprehensive description of the methodological framework, including:framework overview;data preprocessing;feature extraction empowered by multiple medical experts;attention-based Mixture-of-Experts modeling; andsurvival prediction layer with task-specific optimization [file 12916_2026_4865_MOESM1_ESM.docx]

**Detailed Method**

1. **Framework overview**

Figure1 shows the whole pipeline of our workflow. We assembled a multimodal cohort in which every patient has paired PET/CT imaging and corresponding electronic health records (EHR; clinical notes and reports). The pipeline comprises four stages: (i) multimodal data acquisition, (ii) data preprocessing, (iii) feature extraction empowered by multiple medical experts, and (iv) attention-based mixture-of-experts (MoE) modeling followed by a survival prediction layer.

*Multimodal alignment and structuring.* For imaging, we developed an automatic $PET\leftrightarrow CT$ registration algorithm that outputs spatially aligned volumes for each study. For EHR, we implemented rule-assisted, model-based structuring and cleaning to transform free-text clinical reports into sentence-level, time-stamped, de-identified tokens. All intermediate outputs were reviewed by board-certified physicians. For each patient, the final preprocessed bundle is therefore a triplet

$${\mathcal{\mathcal{B}}}_{i}=\left\{ {\tilde{\mathbf{X}}}_{i}^{\mathrm{PET}},{\tilde{\mathbf{X}}}_{i}^{\mathrm{CT}},{\tilde{\mathcal{T}}}_{i} \right\},$$

where the tildes indicate registered (imaging) and structured (text) data.

*Expert-empowered featureization.* We then apply complementary "experts"-pretrained medical vision models with distinct competencies, classical radiomics, and domain-adapted large language models-to extract groups of features from each modality. These groups are designed to be complementary: e.g., discriminative visual semantics (MedCLIP), anatomic delineations (MedSAM), hand-engineered radiomics, and sentence-level clinical semantics (Med-BERT). The resulting patient-level representation is a set of expert feature groups

$$\mathcal{G}_{i}=\left\{ G_{i,e}^{\mathrm{PET}} \right\}_{e=1}^{E_{\mathrm{PET}}}\cup\left\{ G_{i,e}^{\mathrm{CT}} \right\}_{e=1}^{E_{\mathrm{CT}}}\cup\left\{ G_{i,e}^{\mathrm{TXT}} \right\}_{e=1}^{E_{\mathrm{TXT}}}.$$

*Attention-based MoE and risk modeling.* A two-stage Transformer aggregates features within each group and then across groups, producing a compact decision embedding $\mathbf{z}_{i}$. A Cox-type survival head maps $\mathbf{z}_{i}$ to risk scores for overall survival (OS) and progression-free survival (PFS). We further provide interpretability at three levels: (i) attention-rollout heatmaps on PET/CT slices, (ii) expert-level gating weights that reveal which sources drive each prediction, and (iii) clinicopathologic analyses comparing model-defined high- vs low-risk strata.

1. **Data preprocessing**

*PET/CT harmonization and registration.* Volumes were resampled to a common voxel grid and clipped to modality-specific intensity ranges. PET uptake values were converted to standardized uptake value (SUV) and z -scored within a robust body mask.

Rigid-affine registration aligns PET to CT by maximizing normalized mutual information (NMI):

$$\boldsymbol{\theta}=arg\max_{\boldsymbol{\theta}}\mathrm{NMI}\left( \mathbf{X}^{\mathrm{PET}}\circ\mathcal{T}_{\boldsymbol{\theta}},\mathbf{X}^{\mathrm{CT}} \right)-\lambda\|\boldsymbol{\theta}{\|}_{2}^{2}$$

where $\mathcal{T}_{\boldsymbol{\theta}}$ is a 12-DOF affine transform and $\lambda$ regularizes extreme deformations. The registered pair is

$${\tilde{\mathbf{X}}}^{\mathrm{PET}}=\mathbf{X}^{\mathrm{PET}}\circ\mathcal{T}_{\boldsymbol{\theta}}, {\tilde{\mathbf{X}}}^{\mathrm{CT}}=\mathbf{X}^{\mathrm{CT}}$$

For downstream slice-wise processing, each volume is decomposed into ordered axial slices:

$${\tilde{\mathbf{X}}}^{m}=\left\{ \mathbf{x}_{s}^{m} \right\}_{s=1}^{S_{m}}, m\in\{PET,CT\}$$

*EHR structuring and cleaning.* Each clinical report is de-identified and segmented into sentences $\mathcal{T}=\left\{ t_{j} \right\}_{j=1}^{J}$ using a ruleaugmented tokenizer with section detection (e.g., Impression, Findings). We apply negation and temporality tagging ( $\mathrm{neg}\left( t_{j} \right)$, time $\left( t_{j} \right)$ ) and remove boilerplate. The structured set is

$$\tilde{\mathcal{T}}=\left\{ \left( t_{j},neg\left( t_{j} \right),time\left( t_{j} \right) \right) \right\}_{j=1}^{J}$$

1. **Feature extraction empowered by multiple medical experts**

We instantiate three pretrained medical vision experts, $\Phi^{\mathrm{MEDCLIP}},\Phi^{\mathrm{MEDSAM}}$, and $\Phi^{\mathrm{RAD}}$ , and one pretrained medical language expert, $\Psi^{\mathrm{MEDBERT}}$. Each expert’s native output is passed through a one-layer MLP projection head to obtain a unified embedding dimension $d_{u}.$

*Discriminative vision medical expert (MedCLIP) on PET/CT.* For modality $m\in\{PET,CT\}$ and slice $\mathbf{x}_{s}^{(m)}$, we compute a semantic embedding $\mathbf{v}_{s}^{(m,\text{ MEDCLIP) }}=\Phi^{\text{MEDCLIP }}\left( \mathbf{x}_{s}^{(m)} \right)\in{\mathbb{\mathbb{R}}}^{d_{u}}$. This expert excels at high-level visual semantics (e.g., tumor vs background context).

*Segmentation-centric vision medical expert (MedSAM) on PET/CT.* For the same $\mathbf{x}_{s}^{(m)}$, we compute a morphology-oriented embedding $\mathbf{v}_{s}^{(m,\text{ }\text{MEDSAM)}\text{ }}=\Phi^{\text{MED}\text{SAM }}\left( \mathbf{x}_{s}^{(m)} \right)\in{\mathbb{\mathbb{R}}}^{d_{u}}$. This expert captures lesion shape, size, boundaries, and local spatial context.

*Classical vision medical radiomics.* Per slice, standardized radiomic features are extracted and projected to $\mathbf{v}_{s}^{(m,\text{ RAD}\text{)}\text{ }}=\Phi^{\text{RAD }}\left( \mathbf{x}_{s}^{(m)} \right)\in{\mathbb{\mathbb{R}}}^{d_{u}}$.

*Language expert on clinical text (Med-BERT).* Each clinical sentence $t_{j}$ is embedded by a medical LLM, and and projected to the shared space:

$$\mathbf{u}_{j}=\Psi^{\mathrm{MEDBERT}}\left( t_{j} \right)\in{\mathbb{\mathbb{R}}}^{d_{u}}.$$

*Expert groups*. We organize embeddings into expert-specific groups that preserve inductive biases and enable targeted downstream attention:

$$G_{\mathrm{MEDCLIP}}^{(m)}=\left\{ \mathbf{v}_{s}^{(m,MEDCLIP)} \right\}_{s}, G_{\mathrm{MEDSAM}}^{(m)}=\left\{ \mathbf{v}_{s}^{(m,MEDSAM)} \right\}_{s}, G_{\mathrm{RAD}}^{m}=\left\{ \mathbf{v}_{s}^{(m,\text{ RAD}\text{)}\text{ }} \right\}_{s}, G_{\mathrm{MEDBERT}}^{\mathrm{TXT}}=\left\{ \mathbf{u}_{j} \right\}_{j}.$$

1. **Attention-based Mixture-of-Experts modeling**

Our fusion network comprises two symmetric stages: intra-group aggregation followed by inter-group mixture.

*Intra-group aggregation.* For an expert group $G=\left\{ \mathbf{g}_{k} \right\}_{k=1}^{K}$, we first apply a lightweight Transformer encoder $T_{\mathrm{enc}}$with multi-head self-attention. Using scaled dot-product attention,

$$Attn(\mathbf{Q},\mathbf{K},\mathbf{V})=softmax\left( \frac{\mathbf{Q}\mathbf{K}^{\top}}{\sqrt{d}} \right)\mathbf{V}$$

Outputs are pooled by learned attention with a global query $\mathbf{q}_{G}$ :

$$\alpha_{k}=\frac{exp\left( \mathbf{q}_{G}^{\top}\mathbf{W}\mathbf{g}_{k} \right)}{\sum_{l} exp\left( \mathbf{q}_{G}^{\top}\mathbf{W}\mathbf{g}_{l} \right)}, \mathbf{f}_{G}=\sum_{k} \alpha_{k}\mathbf{g}_{k}.$$

This yields one vector per group (e.g., $\mathbf{f}_{\text{MEDCLIP }}^{\text{(m) }},\mathbf{f}_{\text{MEDSAM }}^{\text{(m) }},\mathbf{f}_{\text{RAD }}^{\text{(m) }}$for CT/PET modality $\text{m}$; and $\mathbf{f}_{\text{MEDBERT }}^{\text{TXT }}$for clinical text).

*Inter-group mixture with gating.* Let $\mathcal{\mathcal{F}}=\left\{ \mathbf{f}_{1},\ldots,\mathbf{f}_{M} \right\}$ be all group vectors. A cross-group Transformer produces context-refined tokens $\mathbf{f}_{m}$. A gating network $g(\cdot)$ maps the concatenation $\mathbf{c}=\left[ \mathbf{f}_{1}\|\cdots\|\mathbf{f}_{M} \right]$ to non-negative mixture weights:

$$\mathbf{w}=softmax(g(\mathbf{c})), \sum_{m=1}^{M} w_{m}=1,w_{m}\geq0$$

The final decision embedding is the gated mixture

$$\mathbf{z}=\sum_{m=1}^{M} w_{m}\mathbf{f}_{m}$$

which adaptively emphasizes the experts most informative for the current case. The vector $z$is used as the final decision embedding for each patient.

1. **Survival prediction layer (task-specific optimization)**

We train two independent survival models, one per clinical endpoint. For each task $\tau\in\{OS,PFS\}$, we instantiate a task-specific copy of the fusion module and linear risk head (sharing the same architecture but not parameters across tasks). Given the patient embedding $\mathbf{z}^{(\tau)}$ produced by the task-specific fusion network, the risk score is

$$r^{(\tau)}=\mathbf{w}^{(\tau)\top}\mathbf{z}^{(\tau)}+b^{(\tau)}.$$

Model $(\tau)$ is optimized only on its corresponding survival data $\left\{ T_{i}^{(\tau)},E_{i}^{(\tau)} \right\}$ using the negative Cox partial log-likelihood

$${\mathcal{\mathcal{L}}}_{\text{Cox }}^{(\tau)}=-\sum_{i:E_{i}^{(\tau)}=1} \left[ r_{i}^{(\tau)}-log\sum_{j\in\mathcal{\mathcal{R}}\left( T_{i}^{(\tau)} \right)} exp\left( r_{j}^{(\tau)} \right) \right].$$

At inference, the OS marker $r^{(OS)}$ is produced by the OS-trained model and the PFS marker $r^{(PFS)}$ by the PFS-trained model. Downstream evaluation is conducted separately for each endpoint.
