# Supplementary material for: Interpretable multimodal PET/CT-EHR fusion via mixture-of-experts for prognostic stratification in mantle cell lymphoma: a multicenter study
Source: BMC Med. 2026 Apr 16;24:330. doi: 10.1186/s12916-026-04865-1 (PMC13202896; doi:10.1186/s12916-026-04865-1)
Supplement: Supplementary file 2 — Additional file 2: Tables S1-S2. Table S1—[Univariate analyses of factors predictive of progression-free survival and overall survival in the training cohort.]. Table S2—[The Harrell’s C-index results in the training and validation cohorts] [file 12916_2026_4865_MOESM2_ESM.docx]

**Table S1. Univariate analyses of factors predictive of progression-free survival and overall survival in the training cohort.**

| **Category** | **Variables** | **Progression-free survival** | | | | **Overall survival** | | | |
| --- | --- | --- | --- | --- | --- | --- | --- | --- | --- |
|  |  | **Uni-variate analysis** | | **Multi-variate analysis** | | **Uni-variate analysis** | | **Multi-variate analysis** | |
|  |  | **HR（95% CI）** | **P value*** | **HR（95% CI）** | **P value*** | **HR（95% CI）** | **P value*** | **HR（95% CI）** | **P value*** |
| Clinical parameters | Sex, F/M | 1.495（0.682-3.278） | 0.315 |  |  | 2.988（0.852-10.480） | 0.087 |  |  |
|  | Age,<60/≥60 | 1.180（0.622-2.238） | 0.612 |  |  | 1.453（0.589-3.582） | 0.417 |  |  |
|  | Ann Abor stage, I-II/III-IV | 3.958（0.943-16.611） | 0.060 |  |  | 1.812（0.417-7.876） | 0.428 |  |  |
|  | B symptoms,yes/no | 0.713（0.359-1.418） | 0.335 |  |  | 0.529（0.192-1.459） | 0.219 |  |  |
|  | Splenomegaly,yes/no | 1.083（0.545-2.155） | 0.819 |  |  | 0.566（0.188-1.706） | 0.312 |  |  |
|  | Bulky disease,yes/no | 2.075（0.905-4.760） | 0.085 |  |  | 1.857（0.533-6.465） | 0.331 |  |  |
|  | ECOG PS,≥2/0-1 | 0.515（0.070-3.767） | 0.513 |  |  | 0.971（0.128-7.378） | 0.978 |  |  |
|  | LDH level, elevate/normal | 0.726（0.318-1.655） | 0.446 |  |  | 0.431（0.100-1.867） | 0.260 |  |  |
|  | β2-MG, elevate/normal | 2.028（1.034-3.975） | 0.040 |  |  | 5.470（1.594-18.772） | 0.007 | 5.470（1.594-18.772） | 0.007 |
|  | WBC, elevate/normal | 2.307（1.134-4.695） | 0.021 | 2.460（1.198-5.050） | 0.014 | 1.236（0.412-3.709） | 0.706 |  |  |
|  | Bone marrow involvement,yes/no | 1.105（0.576-2.120） | 0.764 |  |  | 0.421（0.140-1.268） | 0.124 |  |  |
|  | Ki-67,high/low | 2.115（1.101-4.062） | 0.024 | 2.073（1.074-4.001） | 0.030 | 0.960（0.379-2.427） | 0.930 |  |  |
|  | Treatment,BTKi-based/R-CHOP like | 0.778 (0.392-1.546) | 0.474 |  |  | 0.374 (0.106-1.313) | 0.125 |  |  |
| PET metrics | SUVmax, low/high | 1.432（0.507-4.046） | 0.498 |  |  | 2.071（0.474-9.042） | 0.333 |  |  |
|  | TMTV, low/high | 4.290（2.025-9.089） | <0.001 |  |  | 4.616（1.538-13.848） | 0.006 |  |  |
|  | TLG, low/high | 4.345（1.987-9.499） | <0.001 | 4.345（1.987-9.499） | <0.001 | 5.789（1.694-19.782） | 0.005 | 5.789（1.694-19.782） | 0.005 |
| Deep learning | R-signatures, low/high | 27.702（6.650-115.397） | <0.001 | 27.702（6.650-115.397） | <0.001 | 6.862（2.286-20.593） | 0.001 | 6.862（2.286-20.593） | 0.001 |

Abbreviations: CI, confidence interval; SE, standard error; HR, hazard ratio; LDH, lactate dehydrogenase; ECOG PS, Eastern Cooperative Oncology Group performance status; β2-MG, β2-microglobulin; WBC,White blood cell count; SUVmax, maximum standardized uptake value; TMTV, total metabolic tumour volume; TLG, total lesion glycolysis.

*P < 0.05.

**Table S2 The Harrell’s C-index results in the training and validation cohorts**

|  | Training cohort | | Validation cohort | |
| --- | --- | --- | --- | --- |
|  | C-index | 95%CI | C-index | 95%CI |
| Progression-free survival | | | | |
| Multiparametric model | 0.892 | 0.853-0.931 | 0.781 | 0.701-0.861 |
| MIPI | 0.613 | 0.519-0.707 | 0.618 | 0.500-0.736 |
| cMIPI | 0.624 | 0.526-0.722 | 0.633 | 0.506-0.760 |
| Overall survival | | | | |
| Multiparametric model | 0.877 | 0.783-0.971 | 0.862 | 0.768-0.956 |
| MIPI | 0.701 | 0.605-0.797 | 0.603 | 0.448-0.758 |
| cMIPI | 0.688 | 0.590-0.786 | 0.621 | 0.419-0.823 |
